# London as a Place of Study

**Published:** 1903-09-05

**Authors:** 


### The Hospital.

A Journal of

The Medical Sciences and Hospital Administration.

Vol. XXXIV.—No. 884. SEPTEMBER 5, 1903.

### LONDON AS A PLACE OF STUDY.

It must often be a matter requiring anxious consideration, both from intending medical students and from their parents or other responsible advisers, whether the preparation for their future career should be commenced in the metropolis or in one of the great provincial cities. It has become only too apparent of late years that the medical schools of London, as far as the first two years of professional education are concerned, are too numerous, and hence that the pupils are nowhere in sufficient numbers to afford a proper remuneration to teachers of a high class in such subjects as anatomy, physiology, and some other kindred matters, which, after all, must be regarded as furnishing the only foundation upon which a solid superstructure of medical and surgical knowledge can be erected. In provincial cities which contain medical schools this difficulty has been met by a combination among the hospitals, under which they unite for the purpose of affording what may be comprehensively called preliminary instruction, and separate for the purpose of affording clinical instruction. It is manifest that if the same thing could be done on a sufficient scale in London the result might be the establishment of a medical school second to none in the world; a school in which the chair of anatomy, and the chair of physiology, and the chair of organic chemistry would be held as permanent and properly remunerated posts by men of European reputation; while the students would be free, after they had passed their examinations in these subjects, to seek their clinical instruction at any one of the great hospitals, or at more than one if some system of interchange could be established. Something of this kind seems not unlikely to be among the results of the establishment of the new London University; but there are many impediments to be overcome before the conception can be realised. The emoluments of the professors in such a school could not be left to be dependent every year upon the entries of students, and funds for the endowment of the chairs must be provided. Difficulties, however, are made to be overcome by energy and perseverance, and although the central school for the preliminary subjects of medical education is at present no more than a dream it is nevertheless a dream which is likely to be realised at no very distant period.

At the present time the balance of advantage between London and provincial schools is so nicely adjusted that the scale may often be turned towards either by the influence of personal considerations, such as the proximity of relatives or friends, and when this is so it may be safely said that the opportunities of obtaining sound knowledge are everywhere sufficient for those who are determined to use them. We fancy it is also true that the student of a provincial school is more apt than the London student to be imbued with what may perhaps be described as a sort of local patriotism; to be more or less dimly conscious that the credit of the locality from which he comes rests to some extent upon his shoulders, and to be determined to maintain it in a suitable manner. This modified form of provincialism is, of course, not without its drawbacks, and may even tend to the narrowness which springs from membership of a mutual admiration society. When Home's "Douglas" was first presented to a London audience, and was received with the applause it merited, it is recorded that an excited Scot in the gallery shouted out the question, "Whaur's Wully Shakspeare noo?" We have known the published achievements of a provincial surgeon received by his colleagues and pupils with somewhat analogous rapture. We may smile at such enthusiasm, but it is nevertheless not to be despised. It may oftentimes be a powerful incentive to good work, and the errors which it may occasion are seldom other than venial.

But yet, after all, when we have duly praised the energy of the provincial mind, and have paid tribute to the excellence of the provincial schools, London remains on a pinnacle, and in some respects is not only unapproached but unapproachable. There is an element of bigness about the mother city of the British Empire which somehow comes to find expression in her children, or which, at least, appeals to and develops whatever there may be of kindred bigness in their nature. It is only beneath her shadow that the student comes completely into touch with the greatest of those who have gone before him; whose labours have rendered it possible that he, too, should cherish the hope of leaving behind him something which may endure. There are some who regard a qualification as a goal, and to these there is little more to be said. There are others to whom it is but the fulfilment of a condition precedent to their rivalry for the prizes of life; and for these London, at least for a time, is the only appropriate training ground. The ultimate field may be anywhere within the boundaries of the Empire, but the powers and faculties necessary for the struggle should be cultivated at the centre. It is only at the centre that it is easy to acquire a correct sense of relative proportion with regard alike to men and to things, and to escape from the *idola* some of which are as prevalent and deceptive now as they were when Bacon first described them. Moreover, whatever may be the department of work selected, London presents facilities for its pursuit—in the way of authorities, both dead and living, of museums, of laboratories—which cannot be equalled, and the richness and abundance of which are often scarcely so much as suspected. In other words, the mother city is herself a microcosm of the Empire; an epitome alike of its wealth, of its learning, and of its products; and the children who have been trained under her wings will be found, as a rule, always to retain something of the stamp which she alone bestows.

